# Effects of Rubber Hand Illusion and Excitatory Theta Burst Stimulation on Tactile Sensation: A Pilot Study

**DOI:** 10.1155/2020/3069639

**Published:** 2020-04-01

**Authors:** Vanessa N. Frey, Kevin Butz, Georg Zimmermann, Alexander Kunz, Yvonne Höller, Stefan Golaszewski, Eugen Trinka, Raffaele Nardone

**Affiliations:** ^1^Department of Neurology and Neuroscience Institute, Christian Doppler University Hospital, Spinal Cord Injury and Tissue Regeneration Center, PMU, Salzburg, Austria; ^2^Department of Mathematics, Paris Lodron University, Salzburg, Austria; ^3^Karl Landsteiner Institute for Neurorehabilitation and Space Neurology, Salzburg, Austria; ^4^Department of Psychology, University of Akureyri, Akureyri, Iceland; ^5^Department of Neurology, Tappeiner Hospital, Meran, Italy

## Abstract

Synchronous visuotactile stimulation on the own hidden hand and a visible fake limb can alter bodily self-perception and influence spontaneous neuroplasticity. The rubber hand illusion (RHI) paradigm experimentally produces an illusion of rubber hand ownership and arm shift by simultaneously stroking a rubber hand in view and a participant's visually occluded hand. The aim of this cross-over, placebo-controlled, single-blind study was to assess whether RHI, in combination with high-frequency repetitive transcranial magnetic stimulation (rTMS) given as intermittent (excitatory) theta burst stimulation (iTBS) applied over the hand area of the primary sensory region (S1) can enhance tactile sensation in a group of 21 healthy subjects and one patient with cervical spinal cord injury. Four sessions covered all combinations of real and sham stimulations of the RHI and the TBS: real TBS and real RHI, real TBS and sham RHI, sham TBS and real RHI, and both conditions sham. The condition sham TBS and real RHI shows the greatest effect on the proprioceptive drift (median 2.3 cm, IQR 2) and on the score of RHI questionnaires (median 3, IQR 2) in the control group as well as in the real-real condition (median 2, IQR 2). The sham TBS and real RHI condition also shows the best results in the electrical perception test of the patient (median 1.9 mA). Conversely, the upregulation of the cortical excitability of S1 via TBS seems to impair the effect of the RHI. This might be due to a strengthening of the top-down connection between the central nervous system and the periphery, diminishing the RHI. This finding helps in understanding the mechanisms of top-down and bottom-up mechanisms in healthy subjects and patients with spinal cord injury. The RHI paradigm could represent an interesting therapeutic approach in improving tactile sensation and rTMS techniques could modulate these effects. Yet, further studies are needed, to examine the direction of the interaction effect of TMS and RH.

## 1. Introduction

Due to its plasticity, the brain adapts to altered afferent signals in multiple ways, including modification of cortical somatotopy and changed bodily self-perception [1, 2]. One method of testing this feeling of embodiment and the dissociation of oneself to the environment is the rubber hand illusion (RHI) [3]. The RHI paradigm experimentally produces an illusion of rubber hand ownership and arm shift by simultaneously stroking a rubber hand in view and a participant's visually occluded hand [4]. In a typical setup, the participant's hand is hidden and tactile is stimulated while a fake rubber hand is stimulated in the same way visible to the participant. The illusory sensation is evoked via the principle of Bayesian perceptual learning caused by a timely synchronous visual and tactile input [5].

In the past, several studies could demonstrate the effect of the RHI on somatotopy. Schaefer and colleagues [6] showed that the brain in healthy participants adapts immediately to new circumstances after visuotactile manipulation like the RHI. The participants of this magnetoencephalography (MEG) study saw a digit 1 finger being stroked on a digital screen lying in front of the participant on a desk. Synchronously their own digit 5 finger was stroked, hidden from view. The participants reported that they felt the stroking on their first instead of fifth digit as they experienced ownership of the digital hand. The authors observed an increase in source extend of the cortical representation of digit 5 of the somatosensory cortex in the MEG. In another study, the same research group induced the illusion of possessing three hands in healthy participants and observed a shift of the cortical representation of the first digit to a more medial and superior position via MEG [7]. Also, the unusual case of a patient, who had lost upper-left limb sensation after a brachial plexus lesion and developed a phantom sensation of the arm restricted to the ear, has been described [8]. These findings suggest that the sensory homunculus is rather reflecting the perceived shape of the body than a representation caused by physical stimuli.

Moseley and colleagues [9] successfully induced the RHI in healthy participants and measured the skin temperature of their upper and lower extremities. The temperature of the stimulated limb decreased by 0.25-0.27°C, whereas the other limbs that were not affected by the illusion did not change in temperature. The authors stated that the real body part is disconnected from body ownership, and an artificial counterpart is included instead.

However, in another study, temperature differences were not strictly correlated with the subjective feeling of hand ownership in the RHI [10].

Still, this kind of adaptive neuroplasticity can be of advantage in improving the tactile sensation in patients with spinal cord injury (SCI) [11]. A damage of the spinal cord caused by a traumatic or nontraumatic injury is having a great impact not only on the efferent and afferent connections but also on the central nervous system itself [12]. Lenggenhager et al. reported an increase in sensitivity in the fingers of two patients with SCI and sensation in fingers that were previously numb after applying the RHI [11].

In this pilot study, we aimed to explore the effects of an altered body ownership in a healthy control group and in a patient with SCI. We hypothesized that this increased tactile sensitivity can be enhanced via repetitive transcranial magnetic stimulation (rTMS). In fact, high-frequency (HF) rTMS is known to upregulate the cortical excitability [13] and therefore could induce an increase in top-down communication and enhance the connectivity of involved brain areas. Additionally, rTMS is thought to trigger long-term potentials in cortical synapsis, thus influencing long-term plasticity [14].

A well-established rTMS protocol is the so-called theta burst stimulation (TBS), which employs low intensities and has robust, long-lasting effects in normal subjects. Different patterns of TBS induce opposite effects on synaptic efficiency of the stimulated cortex. Intermittent TBS (iTBS) has been shown to increase motor cortical excitability, while continuous TBS (cTBS) decreases cortical excitability [15].

## 2. Material and Methods

### 2.1. Participants

Twenty-one healthy participants were recruited, of which one stopped the study for personal reasons. Three participants had to be excluded from the data analysis, as the RHI could not be elicited. In the final sample, 10 men and 7 women, with a mean age of 29 years (SD 8.6 years) participated. One male patient with nontraumatic SCI (AIS C) was recruited. He was 64 years old and has suffered from an incomplete sub C4 SCI (for 6 month) caused by a Staphylococcus aureus sepsis. The study was approved by the local ethics committee (415-E/2085/4-2016) and all participants signed an informed consent form. The methods were tolerated well by participants and no unexpected events occurred.

### 2.2. Study Design

**Figure 1:**
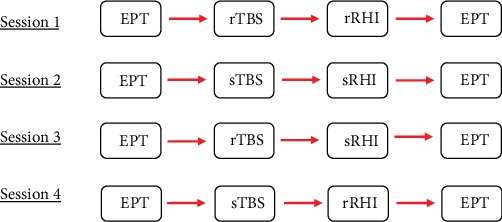
Study design: 4 sessions with a one-week interval. EPT: electrical perception test; rTBS: real theta burst stimulation; sTBS: sham theta burst stimulation; rRHI: real rubber hand illusion; sRHI: sham rubber hand illusion.

This is a cross-over, sham-controlled, single-blind, balanced study. It includes four sessions for every participant, covering all combinations of real and sham stimulations of the RHI and the TBS: real TBS and real RHI (rTBS rRHI), real TBS and sham RHI (rTBS sRHI), sham TMS and real RHI (sTBS rRHI), and both conditions sham (sTBS sRHI). The order of the sessions was balanced and different for every participant. At least a period of one week was given between the single sessions, to exclude a carryover effect of the TBS. At the beginning of each session, the threshold of the participant's tactile sensation was recorded via an electrical perception test (EPT) (before). Then, TBS, the RHI, and another EPT (after) were conducted, respectively.  Figure 1 illustrates the procedure for one possible order of sessions.

### 2.3. Rubber Hand Illusion

The RHI was performed according to the procedure described by Botvinick and Cohens [3]. The participant's hand was placed covered on a desk, while the rubber hand was placed in front of the participant. The experimenter stimulates the real hidden hand and the rubber hand simultaneously for two minutes with two brushes (frequency of approx. 1 Hz), causing the participant to feel the brush touching his hand, while seeing the rubber hand being touched. For the sham condition, the real hand and the rubber hand were stimulated asynchronously (delay of approx. 500 ms).The rubber hand was covered with a medical glove to increase the resemblance as well as the participant's hand. The effect of the RHI was tested via the proprioceptive drift (ppd) in centimeters, quantifying the feeling of the own hand moving towards the rubber hand and via the standardized nine-item questionnaire by Botvinick and Cohen [3] translated into German by the authors. The questionnaires were filled out at every session, just after the RHI. The items let the participants express agreement with statements. The lowest score that can be obtained is 1, which means agreement with the statement, such as perceiving the illusion effectively. The highest score is 7, indicating that the participant totally disagrees with the statement in the questions. Only the first three questions (Q1-Q3) describe the effectivity of the illusion, whereas the other six questions act as the control for suggestibility. Therefore, the focus of our research question lays in Q1-Q3. Question no. 1: “It seemed as if I were feeling the touch of the paintbrush in the location I saw the rubber hand touched.” Question no. 2: “It seemed as though the touch I felt was caused by the paintbrush touching the rubber hand”. Question no. 3: “I felt as if the rubber hand were my hand.”

### 2.4. Prestudy

To exclude a loss in effectivity of the illusion due to desensitization or learning effect during the different sessions, a prestudy on the RHI was conducted. 20 healthy participants, 10 women and 10 men, ranging in age from 18 to 50 years (mean 31 years) each received solely the RHI 3 times with intervals of 2-6 weeks between the sessions. Each application was conducted in the same way like the main study, by the same experimenter. The collected data was statistically analyzed with an ANOVA-type test.

### 2.5. Theta Burst Stimulation

For the TMS, we used the PowerMAG research device by MAG & More. The stimulation pattern was triggered by the software rTMS interface. First of all, the individual resting motor threshold (RMT) and motor hot spot of the first dorsal interosseous (FDI) of the right hemisphere was spotted via single TMS pulses [16].

**Figure 2:**
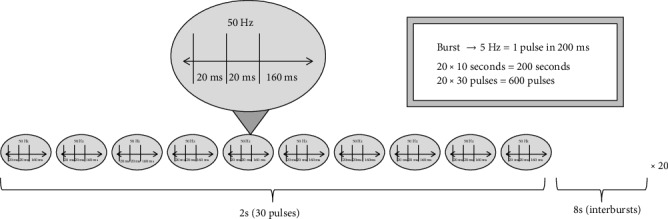
Protocol of intermittent theta burst stimulation.

The primary somatosensory cortex (S1) contralateral to the left FDI was stimulated with the excitatory iTBS. iTBS reaches the maximum facilitating effect due to a higher frequency consisting of three pulses with 50 Hz, given in ten trains for 2 seconds following an 8-second break. This is repeated 20 times, summing up to 600 pulses in 200 seconds (Figure 2) [15]. The intensity was reduced to 80% of RMT.

At the first session, participants wore a bathing cap, on which the nasion and inion were marked, as well as the motor hot spot and the S1 area of the left hand within the right hemisphere (for the S1 cortex the motor hot spot was shifted 2 cm posterior). This cap was used in all the following sessions to make sure the same area on the cortex was stimulated. In the sham condition, the same stimulation protocol was applied, yet the TMS coil was flipped away from the skull at 90 degrees. In the sham TMS and sham RHI condition, sham TMS could not be applied to the patient due to technical problems of the TMS gadget. None of the participants experienced any side effects of the TMS.

### 2.6. Electrical Perception Test

EPT is a well-established, objective, and easily comparable method to test sensation [17]. To test the somatosensory threshold of the hand, an EPT was conducted via the 4-channel EMG System TruTrace by DEYMED Diagnostic. The participant received electrical stimulation (monophasic square wave, lasting 200 *μ*s) via a bipolar electrode on the dermatomes of C6, C7, and C8. Each dermatome was tested for the threshold by increasing the intensity in 0.1 mA steps (from 0 to 2 mA), in 0.2 mA steps (from 2 to 5 mA), and in 0.5 mA steps (from 5 to 10 mA) until the participant felt the stimulation. The intensity was alternately increased and decreased up and down to the threshold (descending and ascending test). Descending and ascending tests were repeated 3 times for each dermatome before and after the RHI and TMS sessions. The location of stimulation on the hand was marked with a pen to make sure there is no variability between the tests at the beginning (before) and end (after) of the sessions.

### 2.7. Statistical Analysis

For the data of the healthy control group, EPT measurements were averaged over three trials. Questionnaire data, EPT measurements, and the proprioceptive drift values were summarized descriptively, using median and quartiles. The data of the patient was not processed statistically. In particular, the statistical significance of discrepancies between healthy controls and the SCI patient could not be assessed, due to *N* = 1 in the latter “group.” Furthermore, due to the exploratory nature of the present investigation, hypothesis tests were conducted for comparisons within the control group. All analyses were performed using R version 3.5.1 (R Core Team 2018).

## 3. Results

### 3.1. Prestudy

**Figure 3:**
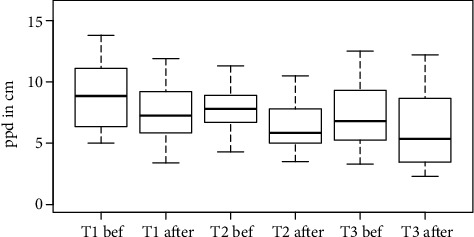
The proporioceptive drift (ppd) in cm for time points T1-T3 before and after the rubber hand illusion. The data is a mean of all participants.

**Figure 4:**
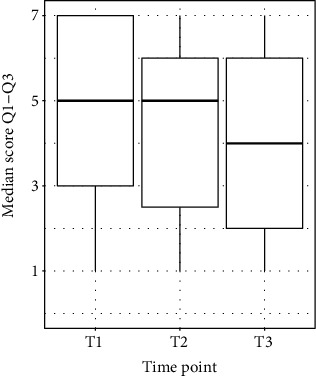
The median scores of the rubber hand illusion questionnaire reached for questions Q1-Q3 for time point 1 (T1), time point 2 (T2), and time point 3 (T3).

The results of the prestudy showed that the RHI was successfully initiated, as the proprioceptive drift was higher after the illusion compared to before (*p* < 0.0001). The data also indicate that there is no difference in the proprioceptive drift (*p* = 0.213) and the answers of the questionnaire (*p* of question 1 = 0.173, *p* of question 2 = 0.677, and *p* of question 3 = 0.985) between the different sessions (Figures 3 and 4); hence, no time trend could be observed by repeating the RHI several times.

### 3.2. Electrical Perception Test

In the healthy control group, there was no difference in the threshold between before and after the TBS and RHI stimulations, in neither C6, C7, nor C8, in none of the examined conditions. In addition, there was no difference between the single conditions (Table 1).

In the SCI patient, there were differences between the thresholds obtained before and after TBS and RHI stimulations (Table 2). When considering all three dermatomes together, the condition sham TBS and real RHI led to the largest improvement in sensitivity (median 1.9 mA), the sham-sham condition exhibited the least improvement (median -3.5 mA), and the real-real condition resulted in a higher improvement than the real TBS and sham RHI condition. According to this, the application of the RHI caused a lowering in the threshold of sensitivity, most effectively without TBS stimulation.

### 3.3. Proprioceptive Drift

**Figure 5:**
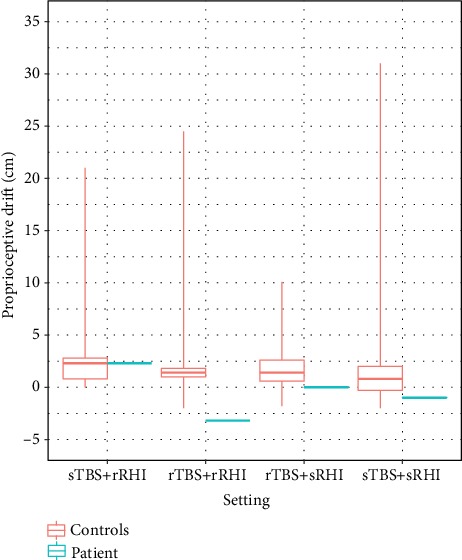
Comparison of the proprioceptive drift of the patient and the healthy control group in centimeters. The whiskers extend to the minimal and maximal data points. rRHI: real rubber hand illusion; sRHI: sham rubber hand illusion; rTBS: real theta burst stimulation; sTBS: sham theta burst stimulation.

In healthy participants, the greatest effect on the ppd was observed in the sham TBS and real RHI condition (median 2.3 cm, IQR 2). In the real-real and real TBS and sham RHI conditions, the median ppd was 1.4 cm (IQR: 0.8 and 2, respectively) and 0.8 cm (IQR: 2.3) in the sham-sham condition (Figure 5).

In the patient, the proprioceptive drift was negative (-3.2 cm) in the real-real condition, meaning the patient subjectively estimated the position of his hand further away instead of closer to the rubber hand. In the condition sham TBS and real RHI, the ppd was most effective (2.3 cm). The conditions with sham RHI do not show either any shift (0 cm) or a negative shift (-1 cm) of the hand (Figure 5).

### 3.4. Questionnaires

**Figure 6:**
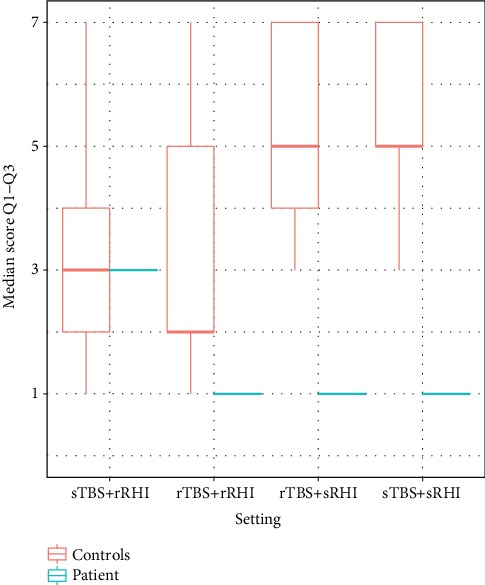
Comparison of the median of the first three questions (Q1-3), between the patient and the healthy control group in score points. rRHI: real rubber hand illusion; sRHI: sham rubber hand illusion; rTBS: real theta burst stimulation; sTBS: sham theta burst stimulation.

Taking together the scores of Q1-Q3, the illusion seems to be most effective in the sham TBS and real RHI and real-real conditions in the control group (Table 3 and Figure 6). The patient subjectively feels the illusion most effective in the sham-sham and real-real conditions; the condition sham TBS and real RHI was rated least effective.

Summing up the results, the condition sham TBS and real RHI showed the greatest effect on the ppd of the patient and control subjects and the EPT in the patient, as well as (together with the real TBS and real RHI condition) on the questionnaire scores in the control group (Table 4).

## 4. Discussion

The findings of this study suggest that the RHI itself caused a great effect, which however seemed to be “disturbed” by excitatory TBS over S1. The results of the questionnaires in the control group met the expectation that the conditions with real RHI are most effective. Since the scores only differed marginally between the real and sham TBS conditions, it seems that TBS did not have an effect on the self-reported subjective intensity of the RHI.

Zeller and colleagues [18] hypothesized that the stimulation of S1 could enhance the bottom-up conduction from the periphery to the cortex in healthy subjects. The stronger this conduction is built-up, the less effective is the RHI. Several TMS studies showed that in healthy participants a significantly reduced cortical excitability, as assessed by means of motor-evoked potential (MEP) amplitudes, accompanies the disembodiment of the real hand during the RHI experience [19, 20]. These findings are consistent with our results showing a reduced RHI effect after the upregulation of cortical excitability induced by iTBS.

The notion that a strong top-down and bottom-up conduction can decrease the effect of the illusion has been discussed by Mussap and Salton [21], which studied body ownership in patients with bulimia and unhealthy body development (excessive exercise and chemical substance use with psychological aspects of negative self-evaluation). Also, in this study, a successfully induced RHI indicates an inherently unstable perceptual body image. It has been reported that patients with schizophrenia experience a stronger RHI, which might be due to distortion of the incoming environmental information, hence a reduced top-down regulation [22, 23].

Interestingly, our data revealed a lower effect of the RHI in healthy participants than in the SCI patient. It has been demonstrated that RHI can reveal plastic phenomena in SCI. Tactile stimuli on the face drives the sense of hand ownership in hand representation-deprived tetraplegics [24]. In another patient with SCI, pain and somatic sensation were found to be transiently normalized by illusory body ownership [25]. Therefore, a correction of the affected body representation could restore a “normal” somatosensory perception and induce analgesic effects that are relevant for patients with SCI. But, a larger sample of SCI patients is needed to confirm this impression and these preliminary findings.

When the S1 and consequently the bottom-up and top-down connections are being upregulated, the RHI itself becomes less effective. These mechanisms represent a possible explanation for the strong effect in the sham TBS and real RHI condition. Therefore, the effect that is described in patients with bulimia and unhealthy body development might be similar to the one in patients with SCI and has not been discussed before in literature.

It should be also considered that during the RHI not only S1 might be highly involved but also a combination of brain areas are likely to be activated, such as the posterior parietal cortex, the ventral premotor cortex, the extrastriate body area [26], temporoparietal junction [27], intraparietal sulcus, and the lateral occipitotemporal cortex [28]. In addition, connectivity between these areas is of great importance. The failed increase of the RHI could be attributed to the fact that only S1 has been stimulated. It would be of great interest in future studies to explore the effects of the stimulation of other areas, alone or in combination. Another limiting factor is the navigation of S1 from the motor hot spot. Ideally, neuronavigation would be used to determine the location of the coil on S1, considering a potential remapping of the somatotopy of the hand.

Even though the ppd is a common method to measure the objective feeling of the RHI, there exist controversial findings. In the opinion of Rhode and colleagues, the ppd does not necessarily represent the effectivity of the RHI [29]. In their study, the drift could also be provoked in the asynchronous stimulation and in a condition without any stroking at all.

According to our data, the sham RHI conditions showed a stronger effect than expected, e.g., in the ppd and the questionnaires in healthy participants. It seems that the rubber hand is integrated well and quickly into the own body perception, while it might actually be the asynchronous sham stimulation that really causes struggle in the feeling of embodiment.

This effect was also observed by Karabanov et al. [30] in their study, as there was a sensory-motor conflict only if the rubber hand was not cooperated in one's body perception. They found that parietal-motor communication shows normal behavior during the RHI; yet, during the sham stimulation, this connection is disturbed by reduced physiological inhibition between the intraparietal sulcus and M1. Zeller and colleagues [29] observed a stronger response in M1 and S1 during the nonillusion condition compared to the illusion condition.

## 5. Conclusion

We have combined two methods, HF rTMS and RHI, aimed at obtaining a supra-additive effect of the RHI and to verify its influence on the tactile sensation in patients with SCI. Our preliminary data included only one patient with SCI and should be interpreted carefully. However, we found that, instead of enhancing the effect of the RHI, HF rTMS seemed to inhibit it, probably by increasing the bottom-up and top-down connections. Hence, the RHI itself may have beneficial effects on improving tactile sensation in patients with SCI. However, an enhancement of this effect might not be possible by stimulating the S1 with excitatory rTMS. It is therefore conceivable that, unlike intermittent TBS, continuous TBS that is known to downregulate the cortical excitability might successfully enhance the RHI and thus increase the improvement of tactile sensation. Since the RHI requires only cheap equipment and does not cause any side effects, it is a promising therapeutic method in patients with impaired sensation. However, due to the exploratory nature of the present investigation, definite conclusions cannot be drawn. Therefore, the effects of RHI and its combination with TMS techniques should be investigated more carefully in future studies.

## Figures and Tables

**Table 1:** Healthy control group: median and interquartile range (IQR) of difference “before minus after” of electrical perception test (EPT) of the different dermatomes C6, C7, and C8 in milliampere. Electrical perception test of different dermatomes in the control group.

	C6	C7	C8
Setting	EPT (mA) (IQR)	EPT (mA) (IQR)	EPT (mA) (IQR)
rTBS rRHI	0.1 (0.4)	0.1 (0.4)	-0.1 (0.7)
rTBS sRHI	-0.1 (0.3)	0.3 (0.6)	0 (0.8)
sTBS rRHI	0 (0.2)	0.1 (0.6)	-0.1 (0.5)
sTBS sRHI	0.1 (0.2)	0 (0.5)	-0.2 (0.7)

rRHI: real rubber hand illusion; sRHI: sham rubber hand illusion; rTBS: real theta burst stimulation; sTBS: sham theta burst stimulation.

**Table 2:** SCI patient: difference “before minus after” of electrical perception test (EPT) of the different dermatomes C6, C7, and C8 in milliampere. Electrical perception test of different dermatomes in the patient.

	C6	C7	C8
Setting	EPT (mA)	EPT (mA)	EPT (mA)
rTBS rRHI	1.2	1.9	0.9
rTBS sRHI	0.1	1.8	-0.3
sTBS rRHI	0.8	1.9	2.3
sTBS sRHI	0.4	-4.7	-3.5

rRHI: real rubber hand illusion; sRHI: sham rubber hand illusion; rTBS: real theta burst stimulation; sTBS: sham theta burst stimulation.

**Table 3:** Questions 1-3 in median score points and interquartile range (IQR) in the control group and the patient.

Control			
Setting	Q1 (IQR)	Q2 (IQR)	Q3 (IQR)
rTBS rRHI	1 (1)	3 (4)	3 (2)
rTBS sRHI	3 (4)	5.5 (2)	5 (3)
sTBS rRHI	1 (1)	3 (3)	4 (3)
sTBS sRHI	5 (4)	5 (3)	6 (2)

Patient			
Setting	Q1	Q2	Q3
rTBS rRHI	1	1	1
rTBS sRHI	1	1	7
sTBS rRHI	1	7	3
sTBS sRHI	1	1	1

rRHI: real rubber hand illusion; sRHI: sham rubber hand illusion; rTBS: real theta burst stimulation; sTBS: sham theta burst stimulation.

**Table 4:** Summary of all results for the patient and the control group.

Condition	ppd		EPT		Questionnaires	
	Control	Patient	Control	Patient	Control	Patient
rTBS rRHI	↑↑	↓↓	→	↑↑	**↑↑↑**	**↑↑↑**
sTBS rRHI	**↑↑↑**	**↑**	→	**↑↑↑**	**↑↑↑**	↑
rTBS sRHI	↑↑	→	→	↑	↑↑	↑↑
sTBS sRHI	↑	↓	→	↓	↑	**↑↑↑**

rRHI: real rubber hand illusion; sRHI: sham rubber hand illusion; rTBS: real theta burst stimulation; sTBS: sham theta burst stimulation; ppd: proprioceptive drift; EPT: electrical perception test. Arrows mean either → no effect, ↓ negative effect, or ↑ positive effect. Numbers of arrows represent the intensity of the effect.

## Data Availability

Original data can be requested via mail: v.frey@salk.at.

## References

[1] Haggard P., Taylor-Clarke M., Kennett S. (2003). Tactile perception, cortical representation and the bodily self. *Current Biology*.

[2] Pazzaglia M., Zantedeschi M. (2016). Plasticity and awareness of bodily distortion. *Neural Plasticity*.

[3] Botvinick M., Cohen J. (1998). Rubber hands ‘feel’ touch that eyes see. *Nature*.

[4] Isayama R., Vesia M., Jegatheeswaran G. (2019). Rubber hand illusion modulates the influences of somatosensory and parietal inputs to the motor cortex. *Journal of Neurophysiology*.

[5] Armel K. C., Ramachandran V. S. (2003). Projecting sensations to external objects: evidence from skin conductance response. *Proceedings of the Royal Society of London. Series B: Biological Sciences*.

[6] Schaefer M., Noennig N., Heinze H. J., Rotte M. (2006). Fooling your feelings: artificially induced referred sensations are linked to a modulation of the primary somatosensory cortex. *NeuroImage*.

[7] Schaefer M., Heinze H.-J., Rotte M. (2009). My third arm: shifts in topography of the somatosensory homunculus predict feeling of an artificial supernumerary arm. *Human Brain Mapping*.

[8] Pazzaglia M., Scivoletto G., Giannini A. M., Leemhuis E. (2020). My hand in my ear: a phantom limb re-induced by the illusion of body ownership in a patient with a brachial plexus lesion. *Psychological Research*.

[9] Moseley G. L., Olthof N., Venema A. (2008). Psychologically induced cooling of a specific body part caused by the illusory ownership of an artificial counterpart. *Proceedings of the National Academy of Sciences of the United States of America*.

[10] Rohde M., Wold A., Karnath H.-O., Ernst M. O. (2013). The human touch: skin temperature during the rubber hand illusion in manual and automated stroking procedures. *PLoS One*.

[11] Lenggenhager B., Scivoletto G., Molinari M., Pazzaglia M. (2013). Restoring tactile awareness through the rubber hand illusion in cervical spinal cord injury. *Neurorehabilitation and Neural Repair*.

[12] Nardone R., Höller Y., Brigo F. (2013). Functional brain reorganization after spinal cord injury: systematic review of animal and human studies. *Brain Research*.

[13] Fitzgerald P. B., Fountain S., Daskalakis Z. J. (2006). A comprehensive review of the effects of rTMS on motor cortical excitability and inhibition. *Clinical Neurophysiology*.

[14] Ziemann U. (2004). LTP-like plasticity in human motor cortex. *Supplements to Clinical neurophysiology*.

[15] Huang Y.-Z., Edwards M. J., Rounis E., Bhatia K. P., Rothwell J. C. (2005). Theta burst stimulation of the human motor cortex. *Neuron*.

[16] Rossini P. M., Burke D., Chen R. (2015). Non-invasive electrical and magnetic stimulation of the brain, spinal cord, roots and peripheral nerves: basic principles and procedures for routine clinical and research application. An updated report from an I.F.C.N. committee. *Clinical Neurophysiology*.

[17] Leong G. W. S., Gorrie C. A., Ng K., Rutkowski S., Waite P. M. E. (2009). Electrical perceptual threshold testing: a validation study. *The Journal of Spinal Cord Medicine*.

[18] Zeller D., Litvak V., Friston K. J., Classen J. (2015). Sensory processing and the rubber hand illusion—an evoked potentials study. *Journal of Cognitive Neuroscience*.

[19] della Gatta F., Garbarini F., Puglisi G., Leonetti A., Berti A., Borroni P. (2016). Decreased motor cortex excitability mirrors own hand disembodiment during the rubber hand illusion. *eLife*.

[20] Kilteni K., Grau-Sánchez J., Veciana De Las Heras M., Rodríguez-Fornells A., Slater M. (2016). Decreased corticospinal excitability after the illusion of missing part of the arm. *Frontiers in Human Neuroscience*.

[21] Mussap A. J., Salton N. (2006). A ‘rubber-hand’ illusion reveals a relationship between perceptual body image and unhealthy body change. *Journal of Health Psychology*.

[22] Ramakonar H., Franz E. A., Lind C. R. P. (2011). The rubber hand illusion and its application to clinical neuroscience. *Journal of Clinical Neuroscience*.

[23] Peled A., Pressman A., Geva A. B., Modai I. (2003). Somatosensory evoked potentials during a rubber-hand illusion in schizophrenia. *Schizophrenia Research*.

[24] Tidoni E., Grisoni L., Liuzza M. T., Aglioti S. M. (2014). Rubber hand illusion highlights massive visual capture and sensorimotor face-hand remapping in a tetraplegic man. *Restorative Neurology and Neuroscience*.

[25] Pazzaglia M., Haggard P., Scivoletto G., Molinari M., Lenggenhager B. (2016). Pain and somatic sensation are transiently normalized by illusory body ownership in a patient with spinal cord injury. *Restorative Neurology and Neuroscience*.

[26] Limanowski J., Blankenburg F. (2016). Integration of visual and proprioceptive limb position information in human posterior parietal, premotor, and extrastriate cortex. *Journal of Neuroscience*.

[27] Tsakiris M., Costantini M., Haggard P. (2008). The role of the right temporo-parietal junction in maintaining a coherent sense of one's body. *Neuropsychologia*.

[28] Limanowski J., Blankenburg F. (2015). Network activity underlying the illusory self-attribution of a dummy arm. *Human Brain Mapping*.

[29] Rohde M., Di Luca M., Ernst M. O. (2011). The rubber hand illusion: feeling of ownership and proprioceptive drift do not go hand in hand. *PLoS One*.

[30] Karabanov A. N., Ritterband-Rosenbaum A., Christensen M. S., Siebner H. R., Nielsen J. B. (2017). Modulation of fronto-parietal connections during the rubber hand illusion. *European Journal of Neuroscience*.

